# Analysis of Ischemia-Modified Albumin (IMA) and Coagulation Parameters in Patients with SARS-CoV-2 Pneumonia

**DOI:** 10.3390/jcm12134304

**Published:** 2023-06-27

**Authors:** Emel Saglam, Gulsen Sener, Tulin Bayrak, Ahmet Bayrak, Numan Gorgulu

**Affiliations:** 1 Department of Internal Medicine, Bagcilar Training and Research Hospital, 34200 Istanbul, Turkey; numangorgulu@gmail.com; 2 Department of Biochemistry, Başakşehir Çam and Sakura City Hospital, 34480 Istanbul, Turkey; drgulsensener@hotmail.com; 3 Department of Biochemistry, Faculty of Medicine, Ordu University, 52200 Ordu, Turkey; bayrakt09@gmail.com (T.B.); abayrak@odu.edu.tr (A.B.)

**Keywords:** COVID-19, ischemia-modified albumin, pneumonia, thrombosis

## Abstract

Background: Coronavirus disease 2019 (COVID-19) is a systemic disease which causes an increased inclination to thrombosis by leading to coagulation system activation and endothelial dysfunction. Our objective in this study is to determine whether ischemia-modified albumin (IMA) can be used as a new marker in patients with COVID-19 for evaluating the increased coagulation risk, pneumonic infiltration, and thus, prognosis. Methods: Our study included 59 patients with COVID-19 compatible pneumonic infiltration on lung computed tomography (CT) who applied to and were hospitalized in the Internal Diseases Outpatient Clinic, then followed up and treated, as well as 29 healthy individuals with a negative COVID-19 rRT-PCR test without any additional disease. Hemogram, coagulation, routine biochemistry, and serum IMA activity parameters were studied. Results: In our study, the higher serum IMA level in COVID-19 patients with pneumonic infiltration compared to that of the healthy control group was found to be statistically significant. No significant correlation was found between the serum IMA levels and the coagulation and inflammation parameters in the 59 COVID-19 patients included. Conclusions: Serum IMA levels in COVID-19 patients with pneumonic infiltration on CT were found to be higher than in the control group. Examination of biochemical parameters, especially thrombotic parameters that affect prognosis such as IMA, can be a guide in estimating pneumonic infiltration.

## 1. Introduction

The human-to-human transmitted coronavirus disease caused by SARS-CoV-2—a new, enveloped, single-stranded RNA-positive Sarbecovirus subgenus β-coronavirus—has been named coronavirus disease 2019 ‘COVID-19’ [1]. COVID-19, emerging in Wuhan, Hubei Province of China in December 2019, sparked an epidemic all over the world, and a global public health emergency arose [2]. On a global scale, as of 21 June 2023, there were 768,187,096 confirmed COVID-19 cases reported to the WHO, including a total of 6,945,714 deaths [3]. By 17 June 2023, as many as 13,398,054,518 vaccine doses were administered [3]. In Turkey, as of 21 June 2023, a total of 17,004,677 confirmed COVID-19 cases with 101,419 deaths were reported to the WHO [4]. By 28 January 2023, a total of 139,694,693 vaccine doses were administered [4].

In the COVID-19 treatment guide, last updated in September 2022, COVID-19 is classified into five different groups based on clinical severity as asymptomatic, mild, moderate, severe, and critical [5]. While mild to moderate infection is observed in 81% of the symptomatic patients, severe disease develops in 14% and critical disease in 5% [6]. While mildly infected patients have a good prognosis, severe and critical patients, in particular, exhibit progress and are hard to treat [7].

The pathophysiology of SARS-CoV-2 is similar to that of SARS-CoV, which damages the respiratory tract with aggressive inflammatory and coagulopathic responses. Uncontrolled inflammation has a critical role in the development of pneumonia and causes damage to organs [8]. Using in vitro diagnostic testing, biochemical monitoring of COVID-19 patients is essential for assessing diagnosis, severity, and progression of disease, as well as rapidly monitoring medical intervention. SARS-CoV-2 leads to the release of some cytokines, including IL-6, by binding to receptors on alveolar and gastrointestinal epithelial cells, and activating the innate and adaptive immune system [9]. The inflammatory response led by excessive release of cytokine seen with T cell and monocyte/macrophage activation encourages vascular permeability, causes exudative fluid accumulation in the alveoli, and leads to respiratory failure [10].

Although the pathophysiology of COVID-19 is rather complex and is not yet totally understood, it has been suggested that the most important factors determining the severity of infection and mortality in an individual infected with SARS-CoV-2 may be age, presence of underlying diseases, presence of secondary infections, viral hyperinflammation, and thromboembolic events [11]. There is a delicate balance between the thrombotic and antithrombotic systems, and when this balance is disturbed, thrombotic conditions emerge. Circulating biomarkers that enable us to detect thrombosis and thromboembolism are probable predictors for the prognosis of patients with COVID-19.

Ischemia-modified albumin (IMA), a type of human serum albumin, where N-terminal amino acids cannot bind transition metals, is a highly sensitive marker for hypoxia [12]. Because of the absence of dyspnea and the extremely low oxygen saturation (sO_2_) level (silent hypoxia) in COVID-19 patients, these patients are at extremely high risk, and the underlying pathomechanism is still unclear [13]. Sanchez et al. observed a significant increase in IMA levels in the early period in 84 patients infected with COVID-19 [12]. This study suggests that serum IMA levels can be taken as an effective new biomarker with high sensitivity for the initial phase of COVID-19 [12].

COVID-19 not only causes many casualties, but also dramatically hampers the overall economy and development [14]. It is very important to define the laboratory tests that can be used to help diagnose and follow COVID-19 patients, tell severe cases from non-severe ones, and determine the risk of mortality.

The early management of thromboembolic complications in COVID-19 patients and the challenges in the early diagnosis for therapeutic intervention still persist. Therefore, new research is needed to develop new biomolecules that will help detect pulmonary involvement and thromboembolic dysfunctions in the early stages of COVID-19.

In studies conducted outside of COVID-19, it has been manifested that the change in serum IMA level is associated with susceptibility to ischemia and thrombosis. We believe there is no current study examining the relationship between IMA and coagulation parameters in COVID-19 patients. Therefore, we examined the correlation of serum IMA values with hemogram and coagulation parameters in patients with COVID-19. In this study, it was aimed to evaluate the relationship between serum IMA level and lung involvement and coagulation in patients infected with SARS-CoV-2.

## 2. Material and Methods

### 2.1. Study Design and Participants

Our study included 59 adult patients over the age of 18 having applied to the Internal Medicine Clinic and were hospitalized in the COVID-19 service, after being diagnosed with COVID-19 via examination, computed tomography (CT) [15], as well as nasal and/or pharyngeal swab samples, with a real-time reverse-transcriptase polymerase-chain-reaction (rRT-PCR) test positivity. Likewise, a total of 29 adults who applied to our hospital between the given dates with negative clinical examination, CT, and rRT-PCR tests without any additional disease were taken as the control group. Informed consent was obtained from the patients. On the first day of their hospitalization, blood samples were taken for hematological and biochemical parameters and were studied in our hospital; the remaining sera were kept at −80 °C for IMA level analysis until they were spectrophotometrically determined.

Patients with hematological disease or who received blood transfusion during their stays in hospital, individuals younger than 18, and pregnant women were excluded from the study.

### 2.2. Clinical Classification

COVID-19 patients were treated in accordance with the Guideline for COVID-19 of the Ministry of Health of Turkey [16]. Our patients were defined as the patients admitted for hospitalization (Group 1) and the controls discharged from the outpatient clinic (Group 2) (within 24 h after admission) [17].

1. Patient group admitted to the service: patients who met the following criteria by confirming the COVID-19 diagnosis through clinical exam, CT, and rRT-PCR test positivity in nasal and/or pharyngeal swab samples.
2. Healthy control group: non-pregnant individuals over the age of 18 who have negative clinical examination, CT, and rRT-PCR tests, and have no additional disease.

One researcher reviewed and scored the thoracic CT images independently with the same CT score criteria: lobar involvement was classified as 0-none (0%), 1-mild (1–50%), 2-moderate (51–75%), or 3-severe (76–100%) of each lobe [15].

The epidemiological, demographic, laboratory, and radiological results and the clinical data of the patients were collected. The parameters and the collected data were compared between the 2 groups.

### 2.3. Biochemical Analysis

The complete blood count and the routine biochemistry and coagulation parameters taken when the patients received their recent diagnosis were examined in our hospital. For the complete blood count, the samples were put into a EDTA tube and the hematology analysis of the samples was carried out with a XN-900 (Sysmex, Kobe, Japan), using absorption photometry and fluorescence flow cytometry.

The routine biochemistry and hormone parameters were put into gel tubes, and the samples were centrifuged at 3500 rpm for 10 min to separate their serum. The samples were studied by the photometric immunoassay method in an Au 480, UniCel DxI 800 (Beckman coulter, Brea, CA, USA). For coagulation parameters, the samples in the citrate tube were centrifuged at 3500 rpm for 10 min and the separated plasma was studied mechanically in an SF-8200 (Succeder, Pekin, China). The IMA activity was analyzed using the serum samples separated from the patients and stored at −80 °C in the biochemistry research laboratory of the Ordu University Faculty of Medicine. The levels of IMA were calculated using Bar-Or et al.’s colorimetric method [18]. A quantity of 200 µL of serum taken from the patient was added to 50 µL of 1 g/L cobalt chloride, mixed and incubated for 10 min in the dark. Then, 2 min after adding 50 µL of dithiothreitol (DTT) solution containing 1.5 mg/mL H_2_O, 1 mL of 0.9% NaCl was included. The absorbance of the mixture was then measured using a spectrophotometer at 470 nm. The results were reported in absorbance units (ABSU).

### 2.4. Ethic

Being a cross-sectional study conducted at the Bagcilar Training and Research Hospital in Turkey, our research was approved by the Faculty of Medicine Ethics Committee of the Ordu University (number: 2020/110, date: 28 May 2020), and informed consent was taken from all participating patients.

### 2.5. Statistical Analysis

For the statistical analysis, NCSS program (Number Cruncher Statistical System) 2007 (Kaysville, UT, USA) was used. In the evaluation phase, descriptive statistical methods and the distribution of the data were evaluated using the Shapiro–Wilk Test. The Kruskal–Wallis Test was used to compare the quantitative data of three or more groups, and the Mann–Whitney U Test to compare two groups. The chi-square test was used to determine the relationship between the qualitative data. Spearman’s rho correlation analysis was preferred to determine the relationship within the quantitative data. ROC analysis was performed with the SPSS program. Significance was evaluated at the levels of *p* < 0.01 and *p* < 0.05.

## 3. Result

A total of 88 patients between the ages of 18 and 95 were studied, and the average age was 48.4 ± 17.2. The average of the IMA values of all of the participants was 0.65 ± 0.12, and the median was 0.39–0.97 (0.64) (Table 1). The other laboratory values are given in Table 1.

In our study, 54.5% (*n* = 48) of the patients were female and 45.5% (*n* = 40) were male, 67% (*n* = 59) were COVID-19 patients and 33% (*n* = 29) were healthy controls. The IMA levels did not show a statistical difference between males and females (Table 2). The IMA value of the COVID-19 patients was significantly higher than that of the control group (*p* = 0.002) (Table 2). The IMA value did not show any statistical significance based on diabetes mellitus type 2 (DM), hypertension (HT), chronic obstructive pulmonary disease (COPD), cardiovascular diseases (CVD), chronic kidney disease (CKD), or cancer (*p* > 0.05) (Table 2). A total of 5 patients had COPD, and 83 had neither restrictive nor obstructive pulmonary disease. No statistically significant correlation was found between CT involvement and COPD (*p* = 0.062), or between sO_2_ % and COPD (*p* = 0.702).

The IMA value for unilateral and bilateral lung infiltration caused by COVID-19 did not show any significance (*p* > 0.05). That the IMA value for the control group was lower than that for the patients with unilateral or bilateral pneumonic infiltration was found significant (*p* = 0.007). The IMA value differed statistically significantly according to CT involvement. It was found statistically significant that the IMA value of the control group was lower than those of the moderate and severe groups (*p* = 0.001) (Table 2).

The mean age of COVID-19 patients was 19–95 (54 year), and the mean age of the control group was 18–68 (37 year) (Table 3). It was found statistically significant that the values for age, glucose, C-reactive protein (CRP), procalcitonin (PRC), aspartate aminotransferase (AST), gama glutamyl transferase (GGT), lactate dehydrogenase (LDH), and ferritin in the COVID-19 group were higher than those in the control group (*p* = 0.001, *p* = 0.003, *p* = 0.001, *p* = 0.001, *p* = 0.002, *p* = 0.026, *p* = 0.001, and *p* = 0.001; respectively) (Table 3). It was found statistically significant that the value for albumin in the COVID-19 group was lower than that for the control group (*p* = 0.001) (Table 3). The urea, creatinine, alanin aminotransferase (ALT), and alkaline phosphatase (ALP) values did not show any statistical significance (*p* > 0.05) (Table 3).

It was found statistically significant that the values for prothrombin time (PT), international normalized ratio (INR), and fibrinogen were higher than those of the control group (*p* = 0.040, *p* = 0.044, *p* = 0.007, and *p* = 0.001; respectively) (Table 4). The values for white blood cell (WBC), hemoglobin (HGB), platelet (PLT), and activated partial thromboplastin time (APTT) based on the groups did not show any statistical significance (*p* = 0.878, *p* = 0.651, *p* = 0.141, and *p* = 0.337; respectively) (Table 4).

It was found statistically significant that the DM and HT were higher in the COVID-19 group than in the control (*p* = 0.009 and *p* = 0.034). There was no significant correlation between the groups and COPD, CVD, CKD, and cancer (*p* > 0.05). No significant correlation was detected between the serum IMA levels and the coagulation and inflammation parameters shown in Table 5 in the 59 COVID-19 patients included in our study (Table 5).

**Figure 1:**
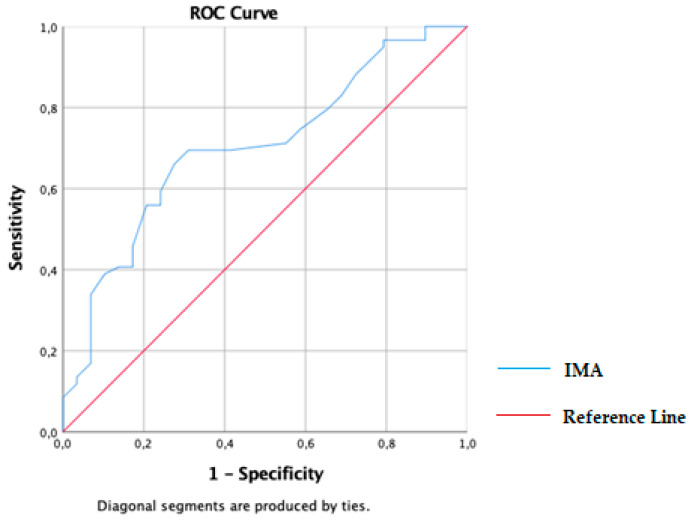
ROC analysis of serum IMA level: Receiver operating characteristic analysis for the best cut-off values of IMA in the patient group.

The sensitivity and specificity were 66.1% and 72.4% when the IMA cut-off was 0.635 (AUC: 70.2). The receiver operating characteristic (ROC) curve showed an area under curve (AUC) value of 70.2 for IMA (Figure 1).

## 4. Discussion

In our study, we found the IMA value in COVID-19 patients and in those with pneumonic infiltration due to COVID-19 was higher than in the control group. The IMA value did not differ in unilateral and bilateral pneumonic involvement of COVID-19 patients. We found the presence of DM and HT in the COVID-19 group was more than in the control. We saw that the serum IMA levels did not correlate with coagulation and inflammation parameters (WBC, HGB, PLT, CRP, PRC, PT, INR, APTT, D-dimer, fibrinogen, and ferritin) in 59 patients with COVID-19.

SARS-CoV-2 infection affects multiple organs, including the lungs, with widespread inflammation through different oxidant and antioxidant biomarkers [19,20]. Hypoxia and tissue hypoperfusion seen in COVID-19 increase the oxidative stress and reactive oxygen radicals (ROS) [21,22]. Related to oxidative balance, serum IMA levels increase when the antioxidant source decreases [23]. In recent studies, higher IMA levels were found in COVID-19 patients compared to control groups [24,25,26]. In Yucel et al.’s study with 70 patients in the ICU, the serum IMA levels were determined to be significantly higher compared to the healthy control group [27]. However, in the study of Altıntas et al., no significant difference was determined in the serum IMA levels between the COVID-19 and the control groups [28]. Many studies have shown that increasing IMA levels contribute to our knowledge about the biomarkers in the pathophysiology of COVID-19, enabling the detection and early diagnosis of pneumonic involvement. In Yıldız et al.’s study, higher levels of IMA were observed in SARS-CoV-2 pneumonia patients compared to the controls [25]. In this study, IMA levels of the patients with severe lung involvement were found to be significantly higher compared to those with non-serious lung involvement [25]. Acar et al.’s study shows that the levels of IMA can be utilized for risk stratification and estimation of mortality in severe COVID-19 pneumonia patients admitted to the emergency unit [26]. In the study of Altıntas et al., no significant difference was found between mild-moderate and severe pneumonia groups and serum IMA levels in COVID-19 patients [28]. In another study, the severity of pneumonia was separated into three groups as mild, moderate, and severe/critical in 95 patients hospitalized due to COVID-19 pneumonia, and although the degree of the disease and the level of IMA increased, statistical significance was not found [29]. In this study, we found that the serum IMA value proved to be higher in the COVID-19 patients and in those with pneumonic infiltration than that of the controls, and that there was no significant correlation between the unilateral and bilateral pneumonic involvement and IMA level.

It is known that additional diseases increase disease severity and hospitalization in COVID-19 patients. In Beaumont et al.’s study conducted on 399 COVID-19 patients, obese or diabetic individuals were found to have a higher risk of hospitalization than those who did not [30]. Studies have shown that the most common comorbidities of COVID-19 are DM, HT, and ischemic heart disease [31,32]. In our study, the higher presence of DM and HT in the COVID-19 patients compared to the controls was found to be statistically significant.

The presence of chronic disease independent of COVID-19 causes an increase in serum IMA level contributing to an increase in ROS and oxidative stress [33]. It was found that the serum IMA level was higher in DM patients than the controls [34]. There are few studies in the literature comparing the presence of additional disease and serum IMA levels in COVID-19 patients. In Yucel et al.’s study, it was found that the levels of IMA were higher in COVID-19 patients with at least one chronic disease than in those without any such diseases [27]. In the study of Ogihara et al., in which they looked at the impact of obesity on disease severity in patients with COVID-19, a higher prevalence of HT and DM was found in the obesity group compared to the non-obese [35]. In this study, although the presence of DM and HT in the COVID-19 group was higher than that in the control group, the IMA value in 59 COVID-19 patients did not differ significantly depending on the presence of comorbidities such as DM, HT, COPD, CVD, CKD, and cancer.

It is known that the inflammation indicators such as CRP, PRC, and ferritin, which are related to the severity of the disease, increase in COVID-19 patients [36,37,38]. In pathophysiological processes associated with increased inflammation and OS, such as COVID-19, albumin is irreversibly oxidized and its level decreases [39,40]. In many studies conducted on COVID-19 patients, it has been shown that the albumin value is low, which may be an indicator of poor prognosis [41,42]. Consistent with the literature, our study found that the CRP, PRC, and ferritin values were higher in the COVID-19 group than in the control group, while albumin was found to be lower.

Various laboratory tests provide guidance to evaluate the course between thrombosis and bleeding seen in the course of COVID-19. Having a high COVID-19 incidence of thrombosis, this condition is associated with the severity of the disease [43]. Increased plasma D-dimer levels in the patients were found to be correlated with the severity of the disease and mortality [43,44]. Patients with COVID-19 pneumonia generally displayed a slight decrease in platelets and an increase in fibrinogen and D-dimer [44,45]. In different studies, PT and aPTT were found to be abnormally shortened [46] or slightly elongated [44] in the patients with COVID-19. In this study, while it was significant that the PT, INR, D-dimer, and fibrinogen values of the COVID-19 group were higher compared to those of the control group, the WBC, HGB, PLT, and APTT values did not show a significant difference.

Since this study is a single-center study with a relatively small sample size, the outcomes cannot be generalized. Secondly, because of the high cost, it was not possible to follow up the ongoing changes in the IMA levels. Future studies with longer follow-up periods and measurements at regular intervals are suggested.

In studies of diseases other than COVID-19, it has been shown that the change in serum IMA level is associated with susceptibility to ischemia and thrombosis. As a result of our literature review, we realized that there is not yet a study evaluating the correlation of IMA and coagulation parameters in COVID-19 patients. No correlation was found between the serum IMA levels and coagulation and inflammation parameters (WBC, HGB, PLT, CRP, PRC, PT, INR, APTT, D-dimer, fibrinogen, and ferritin) in the 59 patients included in our study.

## 5. Conclusions

In conclusion, we demonstrate that serum IMA levels play a role in COVID-19 pneumonia. In our study, for the first time in the literature, serum IMA levels and coagulation parameters were compared in COVID-19 patients affecting the hematopoietic system and they were found to be unrelated. These findings need to be evaluated in further studies with larger numbers of COVID-19 patients.

## Figures and Tables

**Table 1:** Median values for age and laboratory measurements.

	Mean ± SD	Min–Max (Median)
Age	48.48 ± 17.22	18–95 (48.5)
Ischemia-modified albumin (IMA)	0.65 ± 0.12	0.39–0.97 (0.64)
White blood cell (WBC)	8.09 ± 2.98	3.33–20.99 (7.42)
Hemoglobin (HGB)	12.8 ± 1.84	7.7–17.6 (12.85)
Platelet (PLT)	268.32 ± 84.74	102–577 (263)
Glucose	118.19 ± 46.54	74–314 (100)
Urea	36.49 ± 23.61	11.9–149.3 (30.75)
Creatine	0.87 ± 0.68	0.39–5.13 (0.75)
Albumin	3.8 ± 0.63	1.88–5 (3.83)
C-reactive protein (CRP)	38.82 ± 62.13	0.33–315.93 (8.63)
Procalcitonin (PRC)	0.83 ± 5.7	0.01–47.82 (0.04)
Alanin aminotransferase (ALT)	28.23 ± 29.12	6–175 (18)
Aspartate aminotransferase (AST)	29.59 ± 20.9	10–142 (23)
Gama glutamyl transferase (GGT)	34.66 ± 27.89	9–135 (25)
Alkaline phosphatase (ALP)	85.83 ± 34.56	34–248 (80)
Lactate dehydrogenase (LDH)	255.75 ± 106	124–724 (222.5)
Prothrombin time (PT)	13.98 ± 1.3	11.1–19.4 (13.8)
International normalized ratio (INR)	1.09 ± 0.11	0.92–1.49 (1.07)
Activated partial thromboplastin time (APTT)	34.52 ± 4.26	20.1–45.4 (34.6)
D-dimer	0.41 ± 0.59	0.01–3.51 (0.24)
Fibrinogen	430.37 ± 106.5	266–754 (416)
Ferritin	123.85 ± 185.54	4.5–1053 (57.45)

**Table 2:** Comparison analysis based on the IMA value.

Variables		*n*	IMA		*p*
			Mean ± SD	Min–Max (Median)	
Groups	COVID-19	59	0.68 ± 0.11	0.49–0.97 (0.67)	**0.002** ^a^
	Control	29	0.59 ± 0.1	0.39–0.84 (0.59)	
Gender	Male	40	0.66 ± 0.13	0.39–0.89 (0.65)	0.433 ^a^
	Female	48	0.65 ± 0.11	0.49–0.97 (0.64)	
Diabetes Mellitus	Yes	11	0.64 ± 0.08	0.54–0.8 (0.64)	0.875 ^a^
	No	77	0.69 ± 0.12	0.49–0.97 (0.68)	
Hypertension	Yes	8	0.66 ± 0.11	0.54–0.87 (0.64)	0.983 ^a^
	No	80	0.68 ± 0.11	0.49–0.97 (0.67)	
Chronic Obstructive Pulmonary Diseases	Yes	5	0.67 ± 0.1	0.54–0.78 (0.65)	0.620 ^a^
	No	83	0.68 ± 0.11	0.49–0.97 (0.67)	
Cardiovascular Diseases	Yes	3	0.68 ± 0.17	0.54–0.87 (0.64)	0.854 ^a^
	No	85	0.68 ± 0.11	0.49–0.97 (0.67)	
Chronic Kidney Disease	Yes	3	0.65 ± 0.09	0.57–0.74 (0.64)	0.918 ^a^
	No	85	0.68 ± 0.11	0.49–0.97 (0.67)	
Cancer	Yes	2	0.68 ± 0.01	0.67–0.68 (0.68)	0.538 ^a^
	No	87	0.68 ± 0.11	0.49–0.97 (0.67)	
COVID-19 Pneumonic Infiltration	Control	29	0.59 ± 0.1	0.39–0.84 (0.59)	**0.007** ^b^
	Unilateral	12	0.7 ± 0.14	0.49–0.89 (0.73)	
	Bilateral	47	0.67 ± 0.1	0.53–0.97 (0.67)	
Classifications of COVID-19 Pneumonic Infiltration on chest CT	0-none (0%)	29	0.59 ± 0.1	0.39–0.84 (0.59)	**0.001** ^b^
	1-mild (1–50%)	13	0.67 ± 0.13	0.49–0.84 (0.68)	
	2-moderate (51–75%)	34	0.68 ± 0.11	0.53–0.97 (0.67)	
	3-severe (76–100%)	12	0.69 ± 0.1	0.54–0.87 (0.67)	

^a^ Mann–Whitney U test (Mean ± SD), ^b^ Kruskal–Wallis test (Min-Max/Median). All the *p* values that were considered statistically significant (<0.05) are identified in bold.

**Table 3:** Comparison of age and measurements based on the groups.

Variables	Groups	*n*	Mean ± SD	Min–Max (Median)	*p*
Age	COVID-19	59	53.03 ± 17.36	19–95 (54)	**0.001**
	Control	29	39.21 ± 12.8	18–68 (37)	
Glucose	COVID-19	59	129.78 ± 53.89	74–314 (106.5)	**0.003**
	Control	29	98.21 ± 17.3	78–157 (95)	
Urea	COVID-19	59	37.43 ± 25.75	11.9–149.3 (30.35)	0.881
	Control	29	32.58 ± 10.73	17.5–52.3 (32.2)	
Creatinine	COVID-19	59	0.96 ± 0.82	0.39–5.13 (0.78)	0.091
	Control	29	0.7 ± 0.15	0.47–0.96 (0.74)	
Albumin	COVID-19	59	3.6 ± 0.58	1.88–4.86 (3.65)	**0.001**
	Control	29	4.39 ± 0.34	3.57–5 (4.33)	
C-reactive protein (CRP)	COVID-19	59	56.99 ± 69.38	1.34–315.93 (23.1)	**0.001**
	Control	29	5.89 ± 20.11	0.33–110 (1.86)	
Procalcitonin (PRC)	COVID-19	59	1.32 ± 7.22	0.01–47.82 (0.06)	**0.001**
	Control	29	0.02 ± 0.02	0.01–0.09 (0.02)	
Alanin aminotransferase (ALT)	COVID-19	59	30.23 ± 32.42	6–175 (20)	0.304
	Control	29	24.24 ± 21	7–100 (16)	
Aspartate aminotransferase (AST)	COVID-19	59	33.4 ± 24.02	10–142 (25)	**0.002**
	Control	29	22.1 ± 9.21	13–53 (19)	
Gama glutamyl transferase (GGT)	COVID-19	59	38.47 ± 29.82	11–135 (27)	**0.026**
	Control	29	22.08 ± 15.14	9–64 (18)	
Alkaline phosphatase (ALP)	COVID-19	59	90.34 ± 39.17	34–248 (80)	0.334
	Control	29	79.21 ± 25.66	38–148 (78.5)	
Lactate dehydrogenase (LDH)	COVID-19	59	291.48 ± 112.38	162–724 (248.5)	**0.001**
	Control	29	184.29 ± 29.76	124–261 (185.5)	
Ferritin	COVID-19	59	206.63 ± 228.03	6.9–1053 (105.6)	**0.001**
	Control	29	35.36 ± 33.56	4.5–121.8 (22.7)	

Mann–Whitney U Test. All the *p* values that were considered statistically significant (<0.05) are identified in bold.

**Table 4:** Comparison of the hemogram and coagulation parameters based on the groups.

Variables	Groups	*n*	Mean ± SD	Min–Max (Median)	*p*
White blood cell	COVID-19	59	8.34 ± 3.49	3.33–20.99 (7.3)	0.878
	Control	29	7.58 ± 1.41	4.79–9.46 (7.55)	
Hemoglobin	COVID-19	59	12.72 ± 1.82	8.6–17.6 (12.8)	0.651
	Control	29	12.96 ± 1.91	7.7–16.4 (12.9)	
Platelet	COVID-19	59	262.09 ± 93.04	102–577 (255)	0.141
	Control	29	280.79 ± 64.76	158–422 (271)	
PT	COVID-19	59	14.24 ± 1.48	11.1–19.4 (14)	**0.040**
	Control	29	13.65 ± 0.95	12.3–16.6 (13.5)	
INR	COVID-19	59	1.11 ± 0.12	0.92–1.49 (1.09)	**0.044**
	Control	29	1.06 ± 0.09	0.93–1.34 (1.04)	
APTT	COVID-19	59	34.87 ± 5.11	20.1–45.4 (35.1)	0.337
	Control	29	34.13 ± 3.06	28.6–40.8 (33.6)	
D-dimer	COVID-19	59	0.5 ± 0.69	0.08–3.51 (0.25)	**0.007**
	Control	29	0.28 ± 0.37	0.01–1.8 (0.15)	
Fibrinogen	COVID-19	59	476.97 ± 105.07	281–754 (473.5)	**0.001**
	Control	29	375.72 ± 79.86	266–675 (356)	

Mann–Whitney U Test. PT: prothrombin time, INR: international normalized ratio (INR), APTT: activated partial thromboplastin time. All the *p* values that were considered statistically significant (<0.05) are identified in bold.

**Table 5:** Correlation analysis with coagulation and inflammation parameters of the COVID-19 patients.

		1	2	3	4	5	6	7	8	9	10	11	12	13
1. IMA	r	1												
	*p*													
2. Age	r	0.154	1											
	*p*	0.246												
3. WBC	r	0.065	0.013	1										
	*p*	0.630	0.920											
4. Hemoglobin	r	0.125	−0.227	−0.038	1									
	*p*	0.354	0.089	0.777										
5. PLT	r	−0.091	−0.099	0.325 *	0.004	1								
	*p*	0.499	0.460	0.013	0.979									
6. CRP	r	0.085	0.07	0.205	0	0.122	1							
	*p*	0.526	0.602	0.127	0.157	0.365								
7. PRC	r	0.207	0.264	0.265	−0.244	0	0.513 **	1						
	*p*	0.178	0.083	0.086	0.120	0.68	0.000							
8. PT	r	−0.033	−0.096	0.149	−0.184	0.026	−0.045	−0.165	1					
	*p*	0.845	0.572	0.379	0.283	0.881	0.793	0.367						
9. INR	r	−0.055	−0.109	0.156	−0.181	0.019	−0.064	−0.181	0.998 **	1				
	*p*	0.746	0.522	0.355	0.291	0.911	0.711	0.321	0.000					
10. APTT	r	−0.061	0.03	−0.045	−0.480 **	−0.137	0.056	−0.113	0.084	0.094	1			
	*p*	0.734	0.869	0.803	0.005	0.449	0.761	0.566	0.646	0.609				
11. D−dimer	r	0.077	−0.012	0.433 **	−0.305 *	0.201	0.303 *	0.397 *	0.169	0.158	0.266	1		
	*p*	0.615	0.938	0.003	0.047	0.191	0.043	0.018	0.389	0.421	0.208			
12. Fibrinogen	r	0.011	−0.17	−0.073	−0.112	0.029	0.577 **	−0.006	0.01	−0.013	−0.062	−0.058	1	
	*p*	0.951	0.337	0.683	0.536	0.872	0.000	0.975	0.962	0.950	0.770	0.771		
13. Ferritin	r	0.207	−0.233	−0.366 *	0.413 *	−0.415 *	0.05	0.263	−0.265	−0.283	−0.437 *	−0.303	0.129	1
	*p*	0.265	0.206	0.046	0.026	0.023	0.793	0.185	0.221	0.190	0.037	0.141	0.578	

Spearman’s rho. * *p* < 0.05, ** *p* < 0.01. IMA: ischemia-modified albumin, WBC: white blood cell, PLT: platelet, CRP: C-reactive protein, PRC: procalcitonin, PT: prothrombin time, INR: international normalized ratio (INR), APTT: activated partial thromboplastin time.
